# P-1189. Clinical Characteristics and Outcomes of Medically Attended Respiratory Syncytial Virus (RSV) Infections in Children 24-59 months of Age

**DOI:** 10.1093/ofid/ofae631.1373

**Published:** 2025-01-29

**Authors:** Zhe Zheng, Jennifer P King, Linwei Li, Tianyu Sun, Catherine A Panozzo, Thomas G Boyce, Iliana Leony Lasso, Sabine Schnyder Ghamloush, Sonia K Stoszek, Matthew D Snape, Evan J Anderson, Edward Belongia, Huong McLean, Joshua Petrie

**Affiliations:** Moderna, Inc., Cambridge, Massachusetts; Marshfield Clinic Research Institute, Marshfield, WI; Moderna, Inc., Cambridge, Massachusetts; Moderna, Inc., Cambridge, Massachusetts; Moderna, Inc., Cambridge, Massachusetts; Marshfield Clinic Research Institute, Marshfield, WI; Moderna, Inc., Cambridge, Massachusetts; Moderna, Inc., Cambridge, Massachusetts; Moderna, Inc., Cambridge, Massachusetts; Moderna Biotech UK, Inc, Didcot, England, United Kingdom; Moderna, Inc., Cambridge, Massachusetts; Marshfield Clinic Research Institute, Marshfield, WI; Marshfield Clinic Research Institute, Marshfield, WI; Marshfield Clinic Research Institute, Marshfield, WI

## Abstract

**Background:**

RSV infection is a major cause of lower respiratory tract disease (LRTD) among young children. However, clinical features of RSV disease in children ≥24 months of age are not well described. This study assessed the signs, symptoms, and clinical characteristics of RSV in children 24-59 months of age in both outpatient and inpatient settings.

**Figure:**
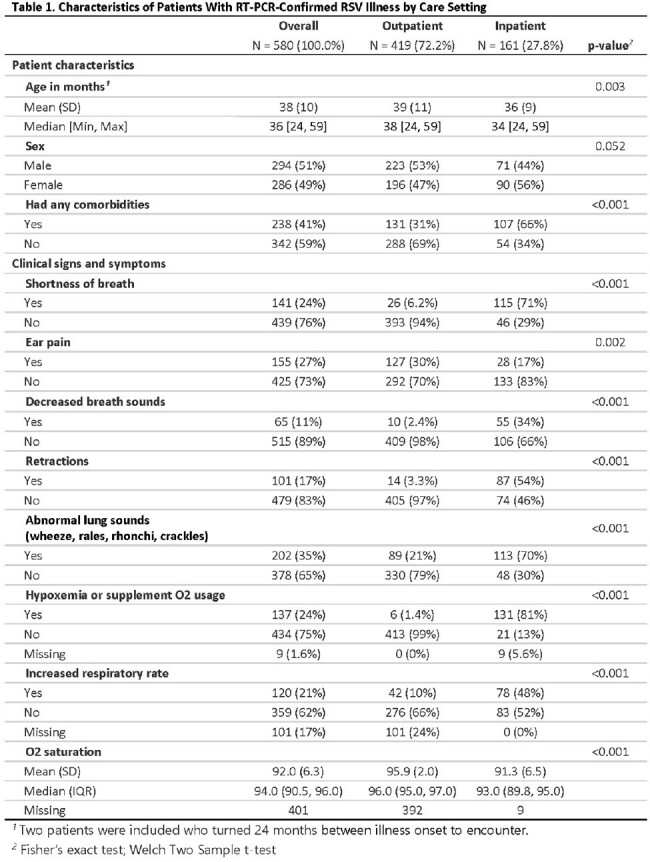

**Methods:**

We abstracted clinical records from RSV-positive children 24-59 months of age who sought outpatient care for acute respiratory illness (ARI) and who had provided a respiratory swab for RSV testing by RT-PCR as part of influenza vaccine effectiveness studies from 2004-10 and 2014-15. Records were also abstracted for hospitalized children with laboratory-confirmed RSV from 2010-23. We analyzed clinical features of RSV in each care setting.

**Results:**

We identified 580 pediatric patients 24-59 months with medically attended, laboratory-confirmed RSV infection, including 419 outpatient and 161 inpatient encounters. The prevalence of comorbidities was 31% among outpatients and 66% among inpatients. The median hospital stay was 3.0 days (IQR: 2.0 - 4.3), and 22% were admitted to the pediatric intensive care unit (PICU). Abnormal lung sounds were observed in 21% of outpatients and 70% of hospitalized patients (P< 0.001). Shortness of breath was documented in 6% of outpatients and in 71% of inpatients (P< 0.001). Increased respiratory rate was recorded in 10% of outpatients and in 48% of inpatients (P< 0.001). The median oxygen saturation at the first measurement for outpatients was 96% (IQR: 95%–97%), compared to 93% (IQR: 90%–95%) for inpatients (P < 0.001). In the complete dataset, 25% of outpatients and 93% of hospitalized children aged 24-59 months exhibited signs or symptoms of LRTD, such as retractions, abnormal lung sounds, hypoxemia and/or need for supplemental oxygen.

**Conclusion:**

Among children 24-59 months of age with medically attended RSV infection, two-thirds of outpatient visits and one-third of inpatient visits occurred in previously healthy children. Nearly one-quarter of hospitalized children required PICU admission, indicating significant morbidity in this age group. Further research on the epidemiology of RSV in children outside of infancy is needed.

**Disclosures:**

**Zhe Zheng, PhD**, Moderna, Inc.: Employee|Moderna, Inc.: Stocks/Bonds (Public Company) **Jennifer P. King, MPH**, GSK Inc.: Grant/Research Support|ModernaTX: Grant/Research Support **Linwei Li, n/a**, Moderna, Inc.: Employee|Moderna, Inc.: Stocks/Bonds (Public Company) **Tianyu Sun, n/a**, Moderna, Inc.: Employee|Moderna, Inc.: Stocks/Bonds (Public Company) **Catherine A. Panozzo, PhD**, Moderna, Inc.: Employee|Moderna, Inc.: Stocks/Bonds (Public Company) **Thomas G. Boyce, MD**, GSK: Grant/Research Support|Moderna, Inc.: Grant/Research Support **Iliana Leony Lasso, n/a**, Moderna, Inc.: Employee|Moderna, Inc.: Stocks/Bonds (Public Company) **Sabine Schnyder Ghamloush, MD**, Moderna, Inc.: Employee|Moderna, Inc.: Stocks/Bonds (Public Company) **Sonia K. Stoszek, PhD**, Moderna, Inc.: Employee|Moderna, Inc.: Stocks/Bonds (Public Company) **Matthew D. Snape, MBBS, MD, FRCPCH, FPM, FMedSci**, Moderna, Inc.: Employee|Moderna, Inc.: Stocks/Bonds (Public Company) **Evan J. Anderson, MD**, Moderna, Inc.: Employee|Moderna, Inc.: Stocks/Bonds (Public Company) **Huong McLean, PhD, MPH**, CSL Seqirus: Advisor/Consultant|CSL Seqirus: Grant/Research Support|GSK: Grant/Research Support|ModernaTX: Advisor/Consultant|ModernaTX: Grant/Research Support **Joshua Petrie, PhD**, CSL Seqirus: Grant/Research Support|Moderna, Inc.: Grant/Research Support

